# Generation of Acid Sites in Nanostructured KIT-6 Using Different Methods to Obtain Efficient Acidic Catalysts for Glycerol Acetalization to Solketal

**DOI:** 10.3390/molecules29235512

**Published:** 2024-11-21

**Authors:** Ewa Janiszewska, Jolanta Kowalska-Kuś, Justyna Wiktorowska, Aldona Jankowska, Agata Tabero, Agnieszka Held, Stanisław Kowalak

**Affiliations:** Faculty of Chemistry, Adam Mickiewicz University, Uniwersytetu Poznańskiego 8, 61-614 Poznań, Poland; jolakow@amu.edu.pl (J.K.-K.); juswik1@st.amu.edu.pl (J.W.); aljan@amu.edu.pl (A.J.); agata.tabero@amu.edu.pl (A.T.); awaclaw@amu.edu.pl (A.H.)

**Keywords:** glycerol acetalization, solketal, KIT-6 synthesis, KIT-6 modification, grafting of sulfonic groups, acidic active centers

## Abstract

This study explored the preparation of pure silica KIT-6, as well as KIT-6 materials with an enhanced concentration of surface OH groups through aluminum incorporation or NH_4_F treatment. These materials with various contents of surface OH groups were subsequently modified via the post-synthesis grafting of sulfonic groups using 3-mercaptopropyltrimethoxysilane as a precursor, followed by oxidation to introduce acidic sites. The catalysts were thoroughly characterized using XRD, nitrogen adsorption/desorption, SEM-EDS, TEM, and FT-IR techniques to confirm their structural and chemical properties. The catalytic activity of acid-functionalized mesoporous silicas of the KIT-6 structure was further evaluated in the acetalization of glycerol to produce solketal. The results demonstrated a significant influence of the surface OH group concentration and acidic site density on catalytic performance, with KIT-6_F_SO_3_H showing the highest efficiency in glycerol-to-solketal conversion. This study provides valuable insights into the design of efficient catalytic systems for the valorization of biodiesel-derived glycerol into high-value chemicals, offering a sustainable approach to waste glycerol utilization.

## 1. Introduction

Ordered mesoporous materials have garnered significant interest due to their promising applications in areas such as catalysis, adsorption, ion exchange, and the development of advanced functional materials [1,2,3,4,5,6,7]. Examples of mesoporous siliceous materials, including MCM-41, SBA-15, SBA-3, and KIT-6, have been synthesized using self-assembly methods with long-chain surfactants as templates. These materials have attracted considerable attention due to their remarkable properties, such as large surface areas, uniform pore sizes, and well-defined pore structures, which further provide an excellent base for numerous functionalization processes, permitting the preparation of many types of heterogeneous catalysts [8]. Among different mesoporous silica structures, KIT-6 stands out with its three-dimensional cubic Ia3d symmetric structure, featuring an interconnected bicontinuous network of channels [9]. Its structure with a cylindrical pore interpenetration system is similar to those in MCM-48 but is characterized by larger mesopores, thicker walls, and enhanced hydrothermal stability [10,11]. This unique 3D channel architecture allows for easy access to guest species, minimizing the risk of pore blockage. However, a notable drawback of pure siliceous KIT-6 is its lack of intrinsic acidity, which is essential for many catalytic processes. To address this limitation, efforts have been directed toward introducing acidic sites into the KIT-6 framework.

One approach involves the incorporation of heteroatoms-like aluminum into the silica structure, aiming to generate acidic centers [12]. However, this method is often complicated by the acidic conditions required for the synthesis of mesoporous materials, which can lead to the dissolution of the heteroatom source and limit its incorporation into the silica network [13]. The second approach is the surface modification of mesoporous materials, which is commonly achieved through two methods: post-synthesis and direct co-condensation [14]. The post-synthesis grafting method involves a two-step process, while the direct co-condensation method allows for the faster preparation of organic–inorganic hybrid materials. The latter typically involves one-step co-condensation between tetraalkoxy silanes (Si(OR′)_4_, R′ = Et, Me) and organoalkoxy silanes (R-Si(OR′)_3_) using the sol–gel process with a structure-directing agent. This method provides several advantages, including a uniform distribution of organic groups and the simplicity of a one-step preparation process. However, the structural order depends on the amount of organic precursor, and the careful selection of organosilanes is necessary to avoid phase separation or bond cleavage. Grafting, on the other hand, is a post-synthesis modification of pre-formed mesoporous support, where organic groups are introduced after surfactant removal. Mesoporous silicas contain a high concentration of surface silanol groups (Si–OH), which serve as anchoring points for organic functionalization. Silylation typically occurs on free (≡Si–OH) and geminal silanol [=Si(OH)_2_] groups, while hydrogen-bonded silanol groups are less accessible to modification. The advantages of post-synthesis grafting include maintaining the structural order of mesoporous materials, flexibility in choosing functional groups, improved hydrothermal stability, and better control of hydrophilic–hydrophobic properties through the selection of organoalkoxy silanes [15,16].

Among the organic–inorganic hybrid mesoporous materials, samples modified with sulfonic groups represent an excellent option for various applications. A frequently used acid functional group is –SO_3_H, with (3-mercaptopropyltrimethoxysilane (MPTMS)) being the most commonly employed precursor. These functional groups can be incorporated during the formation of the silica matrix via the co-condensation method [17] or by attaching it to pre-existing silanol groups on the silica surface through the grafting method [18]. A key step in the preparation of materials using MPTMS as a precursor for sulfonic groups is the oxidation of thiol groups to sulfonic groups. This is often achieved with environmentally friendly oxidants like hydrogen peroxide (H_2_O_2_), which allows the oxidation process to take place under mild conditions, typically at room temperature [19].

The functionalized KIT-6 material, containing acidic sites, shows significant potential for various acid-catalyzed reactions [20,21,22]. One of the most promising applications is the acetalization of glycerol with acetone [23,24,25]. This reaction is especially valuable as it transforms glycerol, a by-product of biodiesel production, into more valuable compounds, thereby enhancing the economic viability of biodiesel processes [26,27]. The key product of glycerol acetalization with acetone is solketal, which serves as an intermediate in the production of pharmaceuticals and other high-value chemicals. The acetalization process is driven by acidic active centers, and extensive research has been devoted to developing efficient and selective catalysts for this reaction. Traditionally, homogeneous catalysts like sulfuric acid have been used due to their strong acidity, but they come with significant drawbacks such as corrosion, environmental harm, and the need for expensive separation processes [28]. To address these challenges, the use of heterogeneous catalysts with acidic sites is essential [29,30,31,32]. These catalysts include heteropolyacids [33,34], mesoporous silicas [35,36], zeolites [37] metal–organic frameworks (MOFs) [38,39], carbon-based materials [40,41], and polymers [42]. The acetalization reaction of glycerol initiates when the catalyst interacts with the carbonyl group of the substrate, either by protonation (in the case of Brønsted acids) or through coordination with a metal site (for Lewis acids). This interaction produces a protonated intermediate, which subsequently reacts with the hydroxyl groups of glycerol to form a hemiketal or hemiacetal. Following water removal, tertiary carbocation is formed. At this stage, the carbocation is attacked by the hydroxyl groups of glycerol, leading to different products depending on the hydroxyl position involved in the attack. Attack by a secondary hydroxyl leads to solketal formation, while attack by a primary hydroxyl results in a six-membered ring isomer. Product selectivity tends to favor solketal formation due to steric hindrance around the primary hydroxyl, making the secondary hydroxyl more accessible for nucleophilic attack [30].

**Figure 1:**
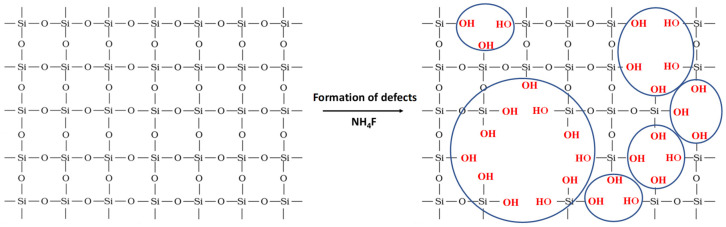
Scheme of the enhancement of the number of OH groups through NH_4_F treatment.

This study focuses on the catalytic performance of KIT-6 materials with enhanced acidic properties for glycerol acetalization with acetone. The goal of the presented studies is to optimize the generation of acid sites through various methods, including synthesizing aluminosilicate KIT-6 and the post-synthesis grafting of sulfonic groups onto the silica structure. Since surface silanol groups are critical anchoring points for sulfonic groups, their concentration directly impacts the final acidic site density in the modified material. Therefore, to enhance the number of surface silanol groups, two approaches were explored: using aluminum-containing KIT-6 and treating pure-silica KIT-6 with the NH_4_F solution (Figure 1).

**Figure 2:**
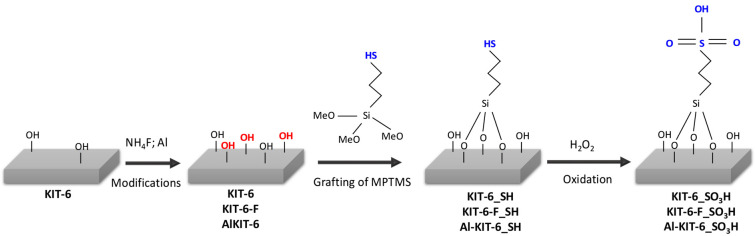
Scheme of grafting of 3-mercaptopropyltrimethoxysilane (MPTMS) and its conversion to sulfonic groups.

Both methods were expected to increase the concentration of OH groups, thereby enhancing the number of -SO_3_H groups introduced during functionalization with 3-mercaptopropyltrimethoxysilane. KIT-6 materials with varying OH group contents were, thus, obtained and further functionalized through the grafting of sulfonic groups (Figure 2).

The catalytic activity of these KIT-6 samples was thoroughly evaluated in glycerol acetalization. The materials with a KIT-6 structure are expected to be superior to mesoporous structures with one- or two-dimensional channels due to the faster diffusion of reactants and products during their reaction in 3D interconnected mesopores. To the best of our knowledge, this is the first comprehensive study to systematically characterize KIT-6 materials with OH groups introduced through various methods and subsequently functionalized with sulfonic groups. This study also establishes a correlation between these structural modifications and their catalytic performance in glycerol acetalization.

## 2. Results and Discussion

### 2.1. Characterization of Catalysts

The synthesis procedure involved obtaining both pure silica and aluminosilicate materials with a KIT-6 structure. It was expected that the introduction of aluminum into the KIT-6 structure during synthesis would generate a negative framework charge, which, similar to zeolites, would be compensated by protons serving as acid sites. Additionally, the presence of aluminum in the reaction mixture might influence the structure formation process of KIT-6, allowing for the production of a material with a different surface OH group content than in the pure silica material. Furthermore, the pure silica material was modified with an NH_4_F solution to create defects containing OH groups in the structure. In this way, KIT-6 structured materials with varying OH group contents were obtained, which were then subjected to a process of grafting sulfonic acid groups onto their surface. The differing content of surface hydroxyl groups is expected to facilitate the introduction of varying amounts of sulfonic groups, which, in turn, could affect the catalytic activity of the resulting materials.

#### 2.1.1. X-Ray Diffraction Measurements (XRDs)

**Figure 3:**
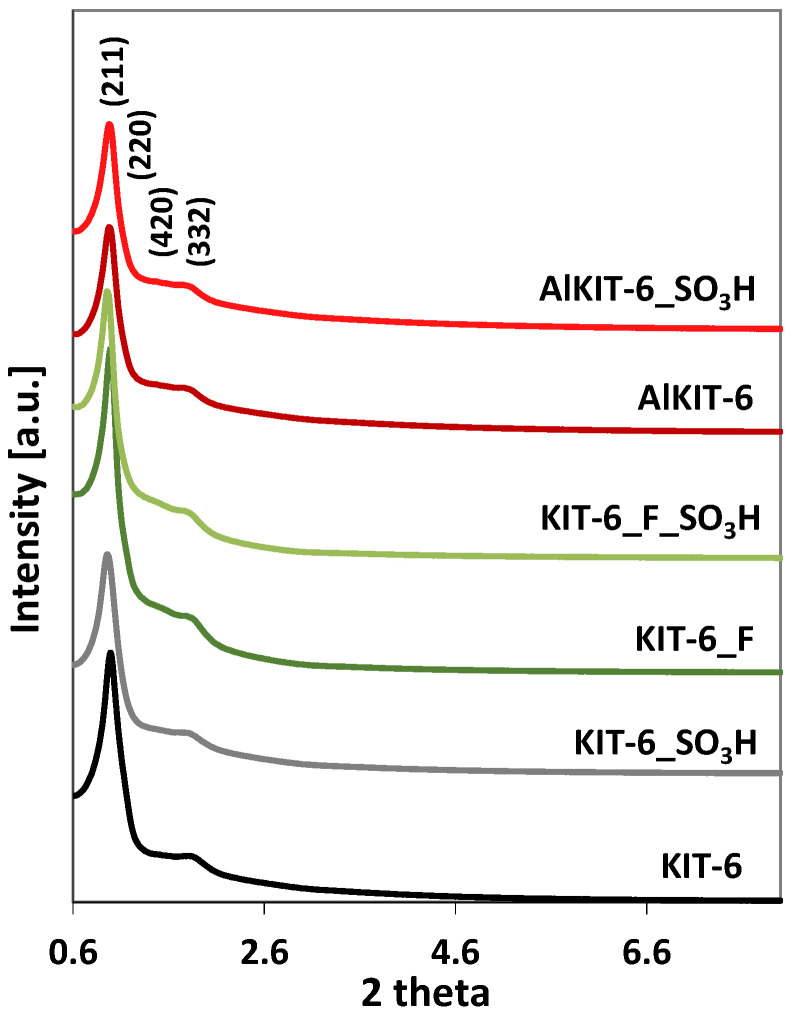
Powder XRD patterns of indicated samples.

X-ray diffraction measurements conducted in the low 2 Θ angle range showed that the obtained KIT-6 materials exhibit a well-ordered structure with Ia3d symmetry, characteristic of KIT-6-type materials [10,43]. Their proper structure is evidenced by the presence of intense reflection at Bragg angle values at around 0.9° and three less intense peaks at 1.1°, 1.5°, and 1.7° corresponding to (211), (220), (420), and (332) planes, respectively (Figure 3).

The introduction of aluminum into the reaction gel enables the production of an aluminosilicate material with a correct KIT-6 structure without disrupting the formation of the ordered mesoporous framework typical of KIT-6. A comparison of the reflection intensities in the X-ray diffractograms of aluminosilicate and siliceous material within the range of 0.6-2.6° reveals a lower intensity for the aluminosilicate relative to the pure siliceous KIT-6. This suggests that while the correct aluminosilicate structure is achieved, it exhibits a lower degree of order compared to the highly ordered structure of the pure siliceous material. A comparison of the X-ray reflections between materials modified with ammonium fluoride and the initial siliceous material showed a slight increase in intensity for the modified samples. This suggests a higher degree of order in the NH_4_F-modified materials, likely due to the removal of disordered domains from the siliceous structure during the modification process. This effect is analogous to the removal of amorphous domains from crystalline zeolites following treatment with fluorine compounds, leading to an overall improvement in structural order [44]. All materials modified with an organosilicate compound containing sulfonic acid groups exhibit lower reflection intensities in their X-ray patterns compared to their non-functionalized counterparts. This decrease in reflection intensity following the attachment of –SO_3_H groups likely indicates a reduction in the degree of structural ordering relative to the original materials.

The unit cell parameter shown in Table 1 was calculated from the formula a_0_ = √6 × d_211_, while the wall thickness (d_s_) was obtained using the formula d_s_ = (a_0_/2) − D (where D is the pore diameter and a_0_ is the unit cell parameter). As can be seen, all modifications (with the introduction of aluminum, treatment with ammonium fluoride, and grafting) affect the wall thickness of the obtained materials. Some modifications (aluminum insertion as well as grafting) also affect the values of the unit cell parameter. The increase in the distances between parallel lattice planes in the case of the AlKIT-6 sample may indicate the incorporation of aluminum into the structure. Since the Al-O bond length is 1.761 Å and the Si-O bond length is 1.603 Å, aluminosilicate materials show higher values of unit cell parameters compared to pure silica materials [45,46,47]. For the sample treated with NH_4_F, we observed a significant decrease in the wall thickness as a result of removing the framework elements. The wall thickness increased slightly after grafting, which may be evidence of the attachment of the organosilicon compound with sulfonic groups to the surface of KIT-6 materials.

#### 2.1.2. Transmission Electron Microscopy (TEM)

**Figure 4:**
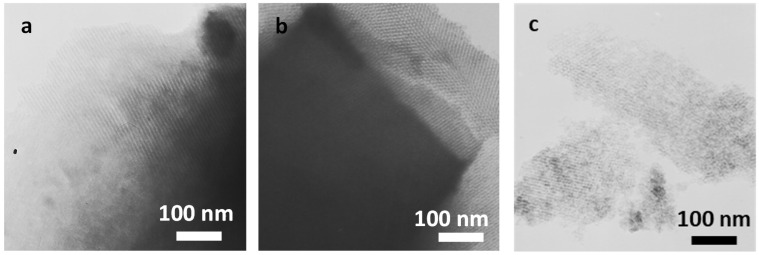
TEM images of KIT-6 (**a**), AlKIT-6 (**b**), and KIT-6_F (**c**).

The ordering of the samples was further confirmed by transmission electron microscopy (TEM). Figure 4 shows a well-ordered cubic array of mesopores of selected samples. The introduction of aluminum atoms to the structure did not affect the ordered structure of the obtained material (Figure 4b). TEM images also revealed no significant agglomerates on the sample’s surface, indicating that the aluminum atoms are well dispersed in the mesopores. Additionally, for the KIT-6 material modified with NH_4_F solution, thinner pore walls were observed, which is confirmed by calculated wall thickness (Figure 4c, Table 1).

#### 2.1.3. Low Temperature Adsorption/Desorption Measurements

**Figure 5:**
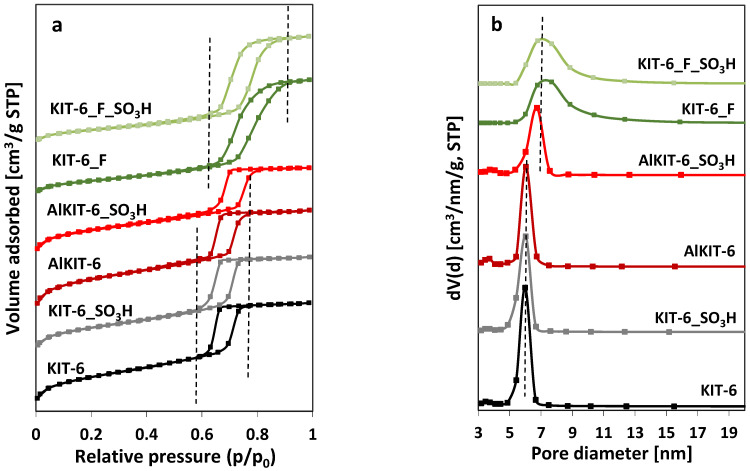
Low-temperature nitrogen sorption isotherms (**a**) and pore size distributions (**b**) for the indicated KIT-6 type materials.

The N_2_ isotherm characterization (Figure 5) offers further insights into the structural order and porosity of the KIT-6 materials. The textural properties, derived from the N_2_ adsorption–desorption isotherm data, are summarized in Table 2. The introduction of aluminum into the KIT-6 structures results in an increase in the specific surface area and total pore volume, accompanied by a reduction in pore diameter. In contrast, the modification of siliceous KIT-6 with NH_4_F solution while maintaining the correct KIT-6 structure leads to a notable decrease in the specific surface area. However, this treatment also causes a significant increase in pore diameter compared to the original KIT-6, likely due to the creation of additional porosity resulting from the removal of silicon atoms from the framework during the modification process. The grafting of sulfonic groups onto the surface of KIT-6 and AlKIT-6 materials significantly reduces their specific surface area (by ~11 and 20%, respectively) and pore volume (by ~6.5 and 14%), which is attributed to the filling of the pores with an organosilicon compound containing sulfonic groups. However, an increase in average pore diameter is observed, indicating the blocking of the smaller pores by the introduced groups—which is a common side effect during surface modification by grafting. For the sample modified with NH_4_F, only a slight decrease in specific surface area (by about 3%) and pore volume (6%) was observed after the grafting process. Similarly to KIT-6 and AKIT-6, an increase in average pore diameter was observed for KIT-6_F_SO_3_H after the functionalization procedure, resulting from the filling of smaller pores with the organosilicon compound.

The nitrogen adsorption–desorption isotherms and pore size distribution are presented in Figure 5. According to the IUPAC classification, all samples exhibit type IV (Figure 5a) isotherms with hysteresis loops, which indicate the presence of ordered mesoporous channels [48]. The hysteresis loops, resulting from capillary condensation within the mesopores, can be classified as H1 type, suggesting that the pores in these materials are cylindrical in shape. The position of these loops (within the relative pressure range of p/p_0_ = 0.6–0.8), as well as their shape and size for silica and aluminosilicate KIT-6 (KIT-6, AlKIT-6) and their functionalized derivatives, aligns with data reported in the literature [49,50]. However, for the NH_4_F-modified sample and its SO_3_H-functionalized form, the loops are broader and shifted to higher relative pressures, indicating larger pore diameters and less uniform pore size, as confirmed by the pore size distributions shown in Figure 5b.

#### 2.1.4. The Content and Density of Hydroxyl and Sulfonic Groups

The determination of weight loss in the temperature range from 773 to 1023 K for unfunctionalized samples enabled the estimation of the number of hydroxyl groups present on the surface of the investigated materials. The results show that both the aluminosilicate sample and the sample modified with NH_4_F have a greater number of surface hydroxyl groups compared to typical siliceous KIT-6 (Table 3). A higher amount of -OH groups on the surface should facilitate the attachment of a greater number of MPTMS molecules, leading to a higher amount of -SO_3_H groups after the oxidation of -SH groups. The sulfonic group content, determined by the back titration of non-exchanged sodium cations, reveals that samples with a higher number of surface hydroxyl groups exhibit a correspondingly greater amount of -SO_3_H groups. The amount of -SO_3_H groups is directly proportional to the number of -OH groups. Furthermore, the calculated -OH group density suggests that in the NH_4_F-modified sample, the hydroxyl groups are more closely spaced compared to those in the KIT-6 and AlKIT-6 samples. This closer proximity may result in the MPTMS molecules attaching via more methoxy groups than on the surfaces of KIT-6 and AlKIT-6 materials, as indicated by the -OH/-SO_3_H molar ratio (Table 3).

#### 2.1.5. FT-IR Spectroscopy

**Figure 6:**
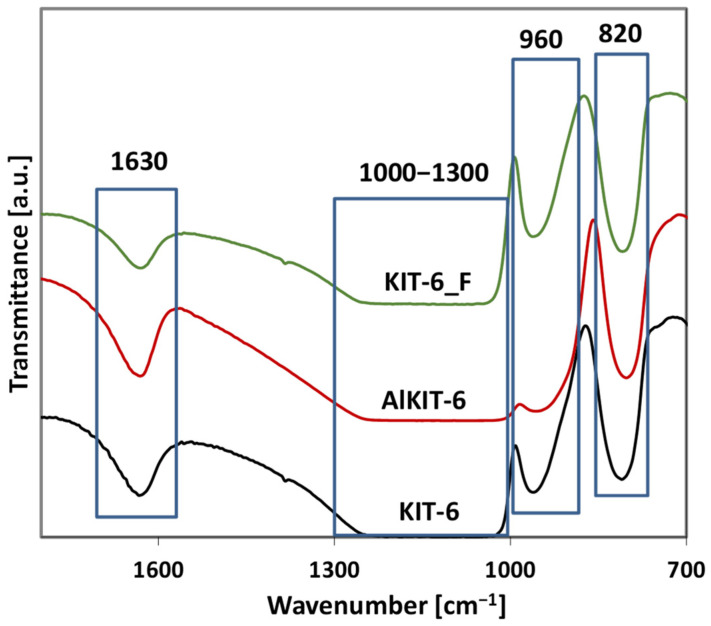
FT-IR spectra of unfunctionalized samples in the framework vibrations range.

The FT-IR spectra of unfunctionalized samples in the range of 700–1800 cm^−1^ reveal intense bands at 1000–1300 cm^−1^, corresponding to the asymmetric stretching vibrations of T–O–T, as well as a band at 800 cm^−1^, attributed to the symmetric stretching vibrations of T–O–T in the amorphous silica framework (Figure 6) [51]. The intensities of these bands are similar across all samples. Additionally, a band at ca. 960 cm^−1^ associated with the symmetric stretching of Si–O bonds of the Si–OH groups was observed, and its intensity can be used as an indicator of the -OH group content in the investigated samples [52]. For aluminum-containing materials, this band shows reduced intensity and notable broadening, which may indicate heterogeneity in the hydroxyl groups, including Si-OH groups and bridging hydroxyl groups. In contrast, for the NH_4_F-modified material, the -OH band is more intense compared to the unmodified siliceous KIT-6. The highest intensity of this band, observed in the NH_4_F-modified sample, is consistent with the hydroxyl group content determined by the thermal method. A band at around 1635 cm^−1^, corresponding to the bending vibrations of OH in adsorbed water molecules, is also observed. In the case of aluminosilicate material, this band shows a slightly higher intensity compared to the unmodified KIT-6. For the fluorine-modified material, this band is less intense, indicating a more hydrophobic character of the materials modified with NH_4_F solutions, which is also confirmed by thermogravimetric analysis. The weight loss for the KIT-6_F sample (recorded on the TG curve) was 5.3%, while for KIT-6, it was 10.4% (Figure S1).

**Figure 7:**
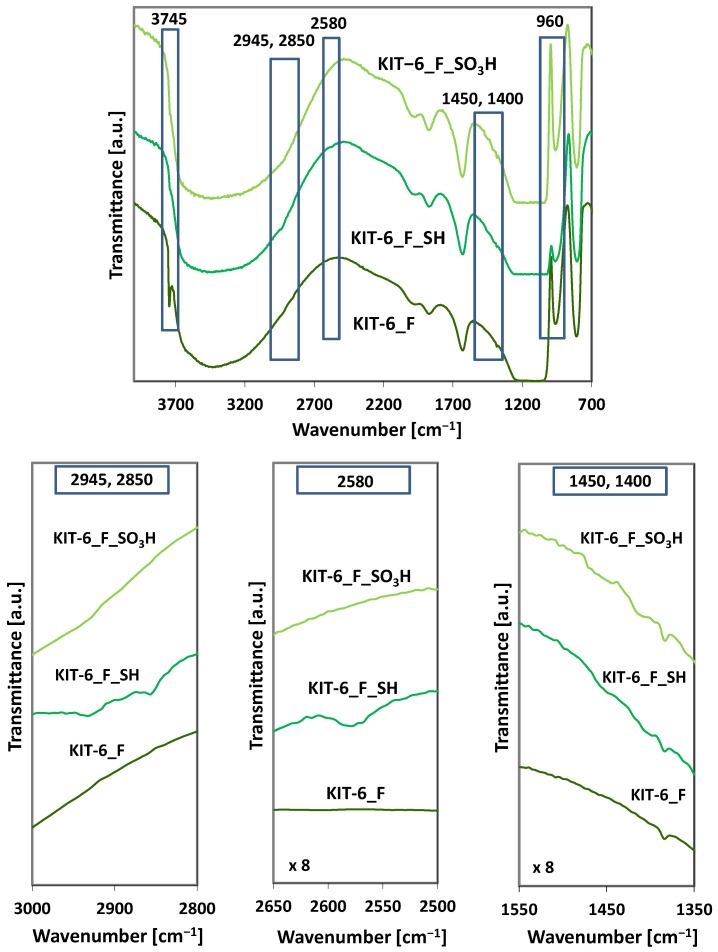
FT-IR spectra of unfunctionalized and functionalized KIT-6_F materials with -SH and -SO_3_H groups.

The spectra of functionalized samples show some differences in comparison to the spectra of unfunctionalized samples (Figure 7).

The absorption band at a wavelength of 3745 cm^−1^ originates from silanol (Si-OH) groups. The absence of this band in the spectra of samples after the functionalization process indicates the disappearance of silanol groups due to their reaction with 3-mercaptopropyltrimethoxysilane. Similarly, the intensity of the band at 960 cm^−1^, also attributed to hydroxyl groups, decreases after the MPTMS modification. This further indicates the involvement of surface -OH groups in binding the modifying agent (MPTMS) to the surface of all functionalized KIT-6 materials. The increase in the intensity of this band after the oxidation of -SH groups is associated with the appearance of -OH groups in the sulfonic groups. The absorption bands observed at wavelengths 2800–2950 cm^−1^ and 1400 cm^−1^ originate from the vibrations of C-H bonds in propyl groups [53] and confirm the presence of mercaptopropyl groups in the functionalized materials (Figure 7). These bands are not very intense due to overlapping with the intense bands originating from water. The band at a wavelength of 2580 cm^−1^ visible in the spectra of the samples with thiol groups originates from the stretching vibrations of -SH bonds. This band disappears upon the oxidation of the -SH groups to -SO_3_H. The bands at ~1200–1250, 1010–1100, and 650 cm^−1^, which correspond to the asymmetric and symmetric stretching vibrations of O=S=O, as well as the stretching vibrations of S-O in sulfonic groups (-SO_3_H), are not observed due to overlap with intense bands originating from silica. The only confirmation of the presence of sulfonic groups after the oxidation of thiol groups is the appearance of the band at 1450 cm^−1^ [54] and the increase in the intensity of the band at 960 cm^−1^. Further evidence of the successful incorporation of well-dispersed sulfonic groups comes from energy-dispersive X-ray spectroscopy (EDS) data corresponding to the SEM images shown in Figure S2. The SEM-EDS spectrum of the AlKIT-6 sample clearly confirms the presence of key elements, including Si, Al, O, and S. Elemental mapping, particularly of sulfur, indicates that the SO_3_H groups are thoroughly and uniformly distributed across the functionalized AlKIT-6 sample.

#### 2.1.6. X-Ray Photoelectron Spectroscopy (XPS)

**Figure 8:**
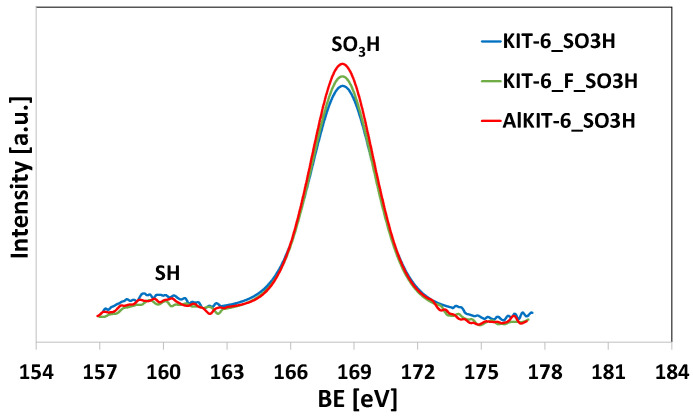
XPS spectra in the S 2p core level region of sulfonated samples.

The successful surface functionalization of KIT-6 materials with sulfur species was also confirmed by XPS measurements (Figure 8). The spectra of the MPTMS-modified samples, following the oxidation of thiol groups, revealed a high-intensity signal at 168.4 eV attributed to sulfonic groups (-SO_3_H) anchored to the silica surface [55]. Additionally, a low-intensity signal at 162.1 eV, corresponding to -SH groups, was also observed, indicating the incomplete oxidation of the thiol groups on the functionalized KIT-6 surface, which is commonly seen in -SO_3_H functionalized materials obtained through post-synthesis oxidation [55,56]. Despite this, sulfonic groups (-SO_3_H) predominated across all samples. Notably, the AlKIT-6_SO_3_H sample exhibited the highest concentration of -SO_3_H groups, while the KIT-6_SO_3_H sample showed the lowest concentration, consistent with the -SO_3_H content determined by back titration (Table 3)_._

#### 2.1.7. UV-Vis Spectroscopy

**Figure 9:**
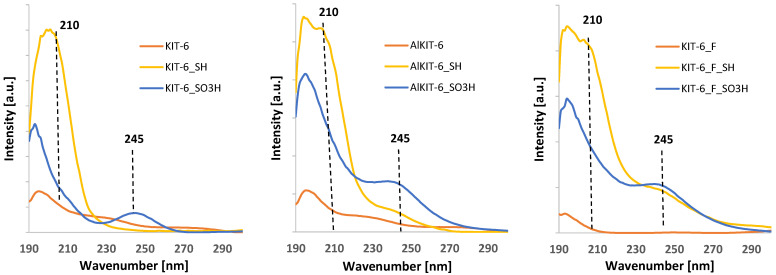
UV-Vis spectra of the indicated samples.

The UV-Vis spectra of siliceous KIT-6, aluminosilicate AlKIT-6, and NH_4_F-modified material are depicted in Figure 9. The modification with MPTMS leads to the appearance of a band at 210 nm in the spectra of all samples. This band is characteristic of electron charge transfer from sulfur atoms present in the mercaptopropyl group [57]. The oxidation of thiol groups results in the disappearance of the band at 210 nm and the appearance of a new band at 245 nm. This newly formed band can be attributed to electron charge transfer within the sulfonic groups, which are generated as a result of thiol group oxidation [58].

The analysis of the UV-Vis spectra also revealed that for AlKIT-6 and KIT-6_F samples after MPTMS modification, in addition to the band originating from thiol groups, a band from sulfonic groups is also visible. This indicates that these samples are susceptible to the oxidation of thiol groups already due to their exposure to atmospheric air.

#### 2.1.8. Acetalization of Glycerol with Acetone

**Figure 10:**
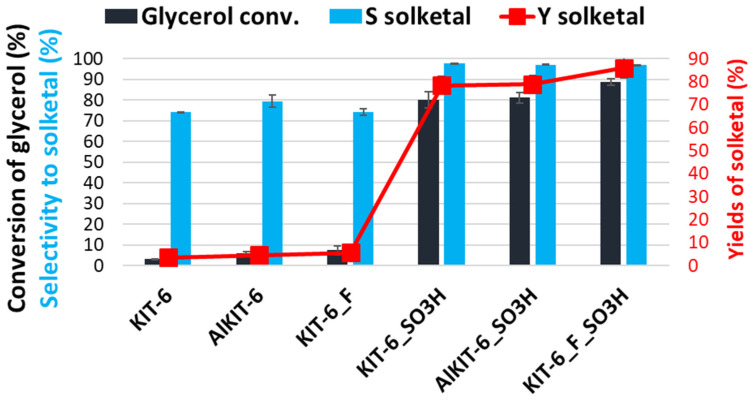
Catalytic activity expressed as glycerol conversion, selectivity to solketal, and yield of solketal for the initial and modified KIT-6 materials and their functionalized analogs in the glycerol acetalization reaction.

The presence of acid sites introduced into the KIT-6 materials was confirmed by their performance as catalysts in a glycerol conversion process as a by-product of biodiesel production. The catalytic activity of all the investigated KIT-6 materials was evaluated in the acetalization of glycerol with acetone, the reaction of which is strongly influenced by the presence of acidic sites. The results of the catalytic activity tests for the materials studied are presented in Table 4 and Figure 10. During the acetalization reaction, the kinetically favored five-membered ring product, known as solketal (2,2-dimethyl-1,3-dioxan-5-ol), is formed, along with the thermodynamically stable six-membered ring isomer (2,2-dimethyl-1,3-dioxolan-4-yl) [59].

Among the tested materials, the pure silica matrix exhibited minimal catalytic activity in the process, with glycerol conversion barely exceeding 3%. This low performance is consistent with the small number of acidic OH sites present in the KIT-6 material (see Table 3). The introduction of aluminum ions into the KIT-6 framework nearly doubled the glycerol conversion, but the result remained unsatisfactory, with conversion reaching around only 6%. A slightly higher glycerol conversion of over 7% was observed for the sample treated with ammonium fluoride. This improvement in glycerol conversion for the Al-KIT-6 and KIT-6_F materials can be attributed to the increased number of acidic sites compared to the original KIT-6 (as shown in Table 3).

**Figure 11:**
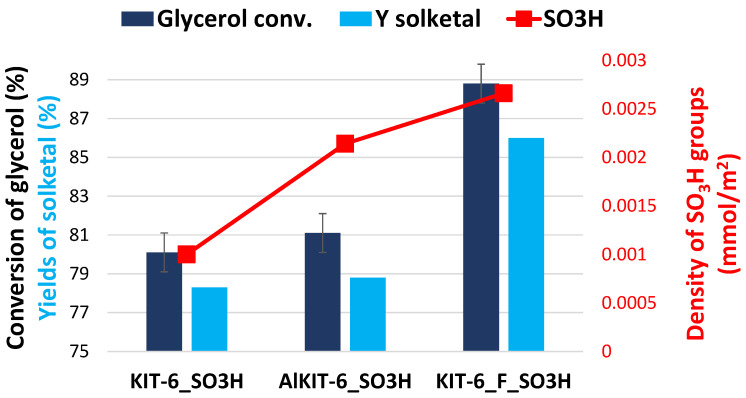
Dependence of glycerol conversion and solketal yield on the density of acidic centers in the glycerol acetalization reaction.

The grafting of sulfonic groups onto all the aforementioned materials significantly enhanced glycerol conversion. The conversion rates across all sulfonated samples ranged from 80% to 89%, though they did not directly correlate with the total number of acidic sites. However, a correlation was observed between glycerol conversion and the density of acidic centers. As the density of acidic centers increased, higher glycerol conversion was achieved (see Figure 11). The highest conversion, reaching 89%, was recorded for KIT-6-F_SO_3_H, which had the greatest density of acidic centers (see Table 3 and Table 4).

**Figure 12:**
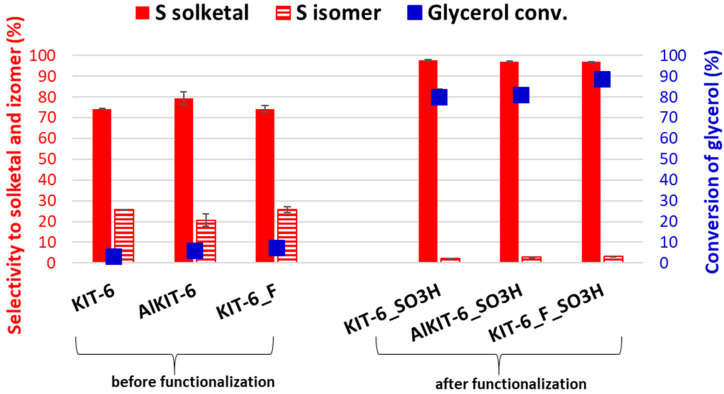
Glycerol conversion and product distribution in the KIT-6 materials before and after the functionalization procedure.

The presence of sulfonic groups not only enhanced glycerol conversion but also significantly altered the product distribution in the acetalization reaction of glycerol with acetone (Figure 12). In the samples that did not contain sulfonic groups, regardless of the modification methods, selectivity towards solketal was noticeably lower, at around 75%, while selectivity towards the six-membered isomer occurred at approximately 25%. However, after grafting sulfonic groups onto the pure KIT-6 matrix as well as its aluminum and NH_4_-modified samples, solketal emerged as nearly the exclusive reaction product, with selectivity ranging between 97 and 98%, while the selectivity towards the six-membered isomer did not exceed 2–3%.

The role of acidic sites, particularly their nature and strength, in glycerol acetalization has been extensively explored in the literature. These studies focus on how acidity affects glycerol conversion and selectivity toward specific products. Both Brønsted and Lewis acid sites are recognized as active centers for catalyzing the acetalization of glycerol [60]. However, Brønsted acidic sites, in particular, are often considered more favorable for the production of solketal, as was emphasized in the work of [61].

Moreover, several authors have pointed out that stronger acidic sites tend to increase selectivity toward solketal. For example, dealuminated BEA zeolites and other catalysts with higher acid strength have been linked to higher selectivity for the formation of five-membered cyclic acetals [59,62]. Additionally, in the presence of strong acidic sites, the six-membered ketal may isomerize to 2,2-dimethyl-1,3-dioxolan-4-yl, leading to an increase in solketal yield, as demonstrated by [63].

Kinetic data presented by Siril et al. [64] suggest that sulfonic acid groups in close proximity can form clusters, where hydrogen bonding between neighboring groups enhances their acid strength. These findings align with the observed differences in selectivity for solketal formation in our studies. The high selectivity to solketal (>97%) in sulfonated samples may be attributed not only to the number of acidic sites but also to the presence of these strong acid clusters. In mesoporous materials like SBA-15, only a small fraction of sulfonic acid groups is close enough to interact and form such clusters. While these clustered arrangements involve only a few sites, they are significantly stronger and can have a disproportionately large impact on catalytic activity. Even a minor concentration of these clustered strong acid sites can substantially enhance catalytic performance. In our functionalized samples, the KIT-6_F_SO_3_H sample exhibited the highest density of sulfonic groups, which explains its superior catalytic activity despite not having the highest overall -SO_3_H content.

## 3. Materials and Methods

### 3.1. Chemicals

The materials used for the synthesis and modification of KIT-6 samples were as follows: Pluronic P123 (Sigma-Aldrich, St. Louis, MO, USA), n-butyl alcohol (Eurochem BGD, Tarnów, Poland), hydrochloric acid (Stanlab, Lublin, Poland), tetraethyl orthosilicate (TEOS, Sigma-Aldrich), aluminum isopropoxide (Al(isop)_3_, Acros Organics, Geel, Belgium), 3-mercaptopropyltrimethoxysilane (MPTMS, Sigma-Aldrich), toluene (Stanlab), H_2_O_2_ (30%, Stanlab), H_2_SO_4_ (96%, Carlo Erba, Cornaredo, Italy), and ethyl alcohol (98%, Stanlab).

The following reagents were used during acid-base titration, which allowed us to examine the presence of acid centers on the surface of functionalized materials: sodium hydroxide (0.1 M, analytical weight, Tarchem, Tarnowskie Góry, Poland) and hydrochloric acid (0.1 M, analytical weight, Chempur, Piekary Śląskie, Poland).

For the acetalization reaction, glycerol (Stanlab) and acetone (Chempur) were applied.

### 3.2. Synthesis of KIT-6 Materials

The synthesis of siliceous and aluminosilicate KIT-6 materials was achieved using P123 as a structure-directing agent and n-butanol as a co-solvent under acidic conditions. The synthesis scheme is presented in Figure S3. The preparation of the mesoporous materials was carried out as follows: 4 g of Pluronic P123 was dissolved in a mixture of 114 mL of distilled water and a 39.2 mL 2 M HCl solution. The solution mixture was subjected to vigorous stirring in a water bath at 313 K until a clear solution was obtained. Then, 6.22 mL of n-butanol was added to the same solution mixture and stirring was continued for one hour. In the next step, 12.84 g of TEOS (and 0.13 g of Al(isop)_3_ in the synthesis of aluminosilicate KIT-6 with an assumed Si/Al ratio of 100 and an actual ratio of 128) was added, and the solution was stirred for 20 h at 313 K. After that, the suspension was transferred into the oven and kept under static conditions at 368 K for 24 h. Then, the solid products obtained were filtered, washed, and dried at room temperature. Finally, the template was removed by the calcination of prepared materials at 823 K for 8 h. The final materials were labeled KIT-6 (siliceous material) and AlKIT-6 (aluminosilicate counterpart).

### 3.3. KIT-6 Modification with NH_4_F Solution

The silica-based KIT-6 material was activated before modification by heating at 623 K for 1 h. For modification, 100 mL of 1 M NH_4_F was added to 1 g of the activated silica. The mixture was stirred and heated under reflux at 333 K for 1 h. Then, the sample was filtered without washing. After drying, the modification procedure was repeated under the same conditions, and the obtained sample was filtered, washed with distilled water, and dried. Finally, the modified silica sample was calcined in a furnace at 623 K for 2 h to convert ammonium ions into protons. The obtained sample was labeled as KIT-6_F. The modification scheme is presented in Figure S4.

### 3.4. Grafting of -SO_3_H Groups onto KIT-6 Materials

Prior to the modification, mesoporous samples were heated at 623 K for 3 h in the oven to remove water from the pores. In total, 1.0 g of anhydrous sample was placed in the round bottom flask equipped with the reflux. Next, 100 mL of anhydrous toluene and 0.33 mL of MPTMS were added. The mixture obtained was heated at 373 K for 5 h, keeping anhydrous conditions. Then, the product was separated by filtration, washed with 100 mL of toluene, and dried at 393 K for 20 h. The amount of added MPTMS was determined by the number of available silanol species on material surfaces, which could react with organosilane, and the amount of MPTMS was added in excess. The number of silanol groups on KIT-6 materials was calculated on the basis of the difference in weight loss between materials calcined at 773 K (the temperature at which adsorbed water was removed from silica) and materials calcined at 1023 K (the temperature at which total dehydroxylation of silica occurs) [65].

The oxidation of grafted thiol groups was carried out using the H_2_O_2_ and H_2_SO_4_ solutions. To perform the oxidation, dry material (dried overnight at 393 K) was immersed in 20 mL of hydrogen peroxide and stirred for 2 h at room temperature. After decantation, the material was washed with 40 mL of an ethanol and water mixture (1:1). Then, the catalyst was immersed in 60 mL of 1 M H_2_SO_4_ and stirred for 2 h at room temperature. Finally, the product was washed with a 20 mL ethanol and water mixture (1:1) and distilled water (until a neutral pH of the filtrate was reached) and dried at room temperature. The scheme of grafting of -SO_3_H groups onto KIT-6 materials is presented in Figure S5.

### 3.5. Characterization

XRD patterns were recorded at room temperature on a Bruker AXS D8 Advance apparatus (Bruker, Billerica, MA, USA) using CuKα radiation (λ = 0.154 nm), with a step of 0.02°.

The FT-IR spectra were recorded using a Bruker Tensor 27 spectrophotometer (Bruker, Billerica, MA, USA). Measurements were performed using the transmission technique in the wavenumber range of 4000–400 cm^−1^ with a resolution of 1 cm^−1^. The sample was mixed with KBr and formed into pellets using a press (150 MPa).

N_2_ adsorption/desorption studies were conducted using a Quantachrome NOVA 1000 instrument (Quantachrome, Boynton Beach, FL, USA). Prior to the measurement, the samples were outgassed at a temperature of 393 K for 16 h. The specific surface area was determined using the BET method, whereas the external surface area and micropore volume were calculated by the t-plot method. The total volume of pores was assessed using the single-point model (at p/p_0_ = 0.99). The mesopore volume was calculated as the difference between the total and micropore volumes (V_tot_–V_micro_). The BJH pore size distributions were derived from the desorption branch.

UV–Vis spectra were recorded using a Jasco V670 UV–Visible spectrophotometer. The spectra were recorded at room temperature in the range from 900 to 190 nm. BaSO_4_ was used as reference material.

Scanning electron microscopy (SEM) studies were conducted using a Quanta FEG 250 (FEI) microscope (Thermo Fisher Scientific, Eindhoven, the Netherlands) under high vacuum conditions with a beam accelerating voltage of 10 kV. Energy dispersive spectroscopy (EDS) analyses were performed at the same voltage using an Octane SDD (EDAX) EDS detector.

Transmission electron microscopy (TEM) images were recorded on a JEOL 2000 microscope (Jeol, Tokyo, Japan) operating at an accelerating voltage of 80 kV.

Thermogravimetric analysis (TG) was performed using a SetSYS 1200 device (Setaram, Geneva, Switzerland). The measurements were performed in a nitrogen atmosphere by heating the sample to a temperature of 500 K with a ramp rate of 10 K/min.

The amount of –SO_3_H groups on the surface of the functionalized materials was determined through ion exchange, where protons from the sulfonic groups were exchanged with sodium ions from a NaOH solution, followed by back titration. A 100 mg sample of the material, dried overnight at 393 K, was soaked in 5 mL of 0.1 M NaOH solution, and then 45 mL of demineralized water was added to the mixture. The mixture was left at room temperature for 24 h. In the next step, the filtrate was separated from the material by centrifugation and further filtered using a syringe filter. For the analysis of sodium ions that did not undergo ion exchange, 20 mL of the filtrate was taken. To this solution, 10 mL of demineralized water was added. The prepared sample was then titrated with 0.1 M HCl using phenolphthalein as an indicator. The number of sulfonic groups was calculated from the difference in sodium ions before and after ion exchange and recalculated per 1 g of the sample. The exchange and titration procedures were repeated two times for each sample, and the result was averaged.

X-ray photoelectron spectroscopy (XPS) measurements were conducted using an Ultra High Vacuum (UHV) System (Specs, Berlin, Germany). The examined materials were irradiated with monochromatic Al Kα radiation (1486.6 eV). The operating pressure in the chamber was close to 2 × 10^−9^ mbar. Binding energies (BEs) were calibrated against the C1s peak set at 284.5 eV. Spectroscopic data were processed by CasaXPS software ver. 2.3.17PR1.1 (Casa Software Ltd., Teignmouth, UK) using a peak-fitting routine with a Shirley background.

### 3.6. Catalytic Tests

The catalytic activity of the samples was examined for glycerol acetalization with acetone. Before the reaction, the catalysts were pre-activated in the air at 473 K for 2 h. The activated materials (0.01 g, 1 wt. % referred to as glycerol mass) were introduced into the glass vials with the equimolar mixture of glycerol (1 g, 11 mmol) and acetone (0.8 cm^3^, 11 mmol). The vials were closed and heated on a magnetic stirrer (400 rpm) at 343 K for 1 h. The reaction products (solketal, the 5-membered ring ketal and isomer, the 6-membered ring ketal), as well as unreacted glycerol and acetone, were identified by mass spectroscopy (Varian 4000 GC-MS, Agilent Technologies, Santa Clara, CA, USA). The results of the reactions were measured by GC analyses on a VARIAN CP-3800 chromatograph (Agilent Technologies, Santa Clara, CA, USA) equipped with an FID detector and a VF-5ms column. Toluene was used as an internal standard. The conversion of glycerol (denoted as Glycerol conv.), selectivity to products: solketal (denoted as S solketal), and isomer (denoted as S isomer), solketal yield (denoted as Y solketal) were calculated according to the equations presented elsewhere [24] and are provided in the Supplementary Materials (Figure S6).

## 4. Conclusions

Ordered mesoporous KIT-6 materials with enhanced acidity were successfully prepared using several methods, including aluminum incorporation, acid site generation through NH_4_F treatment, and post-synthesis grafting with 3-mercaptopropyltrimethoxysilane (MPTMS) with the subsequent formation of -SO_3_H groups to create organic–inorganic hybrid catalysts with acidic sites. The modified KIT-6 materials were then employed as catalysts for glycerol acetalization. The structural integrity of the KIT-6 framework in all samples was confirmed through XRD, nitrogen adsorption/desorption, and the microscopic technique (TEM). The introduction of additional hydroxyl groups on the surface of KIT-6 materials, either through aluminum incorporation or silicon removal, had no significant effect on the activity in glycerol acetalization. The key factor in enhancing activity and selectivity towards solketal was the introduction of strongly acidic sulfonic groups. A strong correlation was observed between glycerol conversion, selectivity to solketal, and the density of acidic centers in the materials. Among the catalysts, the hybrid material KIT-6_F_SO_3_H demonstrated the highest catalytic efficiency, exhibiting superior performance in glycerol valorization.

## Figures and Tables

**Table 1:** The unit cell parameter (a_0_), d-spacing values (d_211_), and the wall thickness (d_s_) for obtained materials.

Sample	d_211_ [nm]	a_0_ [nm]	d_s_ [nm]
KIT-6	8.88	21.7	6.18
AlKIT-6	9.06	22.2	6.89
KIT-6_F	8.88	21.7	1.08
KIT-6_SO_3_H	9.25	22.6	6.53
AlKIT-6_SO_3_H	9.64	23.6	7.21
KIT-6_F_SO_3_H	9.25	22.6	1.43

**Table 2:** Surface area (BET), pore volume, and average pore diameter of obtained KIT-6 materials.

Sample	S_BET_ [m^2^/g]	S_micro_ [m^2^/g]	S_ext_ [m^2^/g]	V_tot_ [cm^3^/g]	V_micro_ [cm^3^/g]	D [nm]
KIT-6	783	142	641	0.92	0.063	4.7
AlKIT-6	881	230	651	0.93	0.108	4.2
KIT-6_F	389	56	333	0.96	0.024	9.8
KIT-6_SO_3_H	695	112	583	0.86	0.051	5.0
AlKIT-6_SO_3_H	700	184	516	0.80	0.085	4.6
KIT-6_F_SO_3_H	376	44	332	0.90	0.021	9.9

S_BET_—specific surface area, S_micro_—micropore surface, S_ext_—external surface, V_tot_—total pore volume, V_micro_—micropore volume, D—pore diameter.

**Table 3:** The content and density of hydroxyl and sulfonic groups for the indicated KIT-6 type materials.

Sample	Content of OH Groups * [mmol/g]	Density of OH Groups * [mmol/m^2^]	Content of SO_3_H Groups [mmol/g]	Density of SO_3_H Groups [mmol/m^2^]	-OH/-SO_3_H Molar Ratio
KIT-6_SO_3_H	1.33	1.70 × 10^−3^	0.75	1.0 × 10^−3^	1.77
AlKIT-6_SO_3_H	2.16	2.45 × 10^−3^	1.50	2.14 × 10^−3^	1.44
KIT-6_F_SO_3_H	2.12	5.45 × 10^−3^	1.00	2.66 × 10^−3^	2.12

* Estimated before the introduction of the SO_3_H groups.

**Table 4:** The results of the catalytic activity expressed as glycerol conversion, selectivity to the solketal and isomer, and the yield of solketal of the original KIT-6 material and its modified and functionalized analogs in the glycerol acetalization reaction.

Sample	Conversion [%]	S_solketal_ [%]	S_isomer_ [%]	Y_solketal_ [%]
KIT-6	3.3	74.3	25.7	2.6
AlKIT-6	5.9	79.5	20.6	4.6
KIT-6_F	7.4	74.3	25.8	5.6
KIT-6_SO_3_H	80.1	97.8	2.2	78.3
AlKIT-6_SO_3_H	81.1	97.1	2.9	78.8
KIT-6_F_SO_3_H	88.8	96.9	3.1	86.0

## Data Availability

The data presented in this study are available on request from the corresponding author.

## Supplementary Materials

The following are available online at https://www.mdpi.com/article/10.3390/molecules29235512/s1: Figure S1. TG analysis of pure silica KIT-6 and NH_4_F-modified (KIT-6_F) material. Figure S2. SEM micrographs, elemental mapping, and EDS data of AlKIT-SO_3_H material. Figure S3. Scheme of KIT-6 and AlKIT-6 synthesis. Figure S4. Scheme of KIT-6 modification with NH_4_F. Figure S5. Scheme of grafting of 3-mercaptopropyltrimethoxysilane (MPTMS) and its conversion to sulfonic groups. Figure S6. Calculation of the catalytic activity results based on chromatographic data.
