# Supplementary material for: Generation of Acid Sites in Nanostructured KIT-6 Using Different Methods to Obtain Efficient Acidic Catalysts for Glycerol Acetalization to Solketal
Source: Molecules. 2024 Nov 21;29(23):5512. doi: 10.3390/molecules29235512 (PMC11643615; doi:10.3390/molecules29235512)
Supplement: Supplementary file 1 [file molecules-29-05512-s001.zip › molecules-3297910-supplementary.pdf]

Generation of acid sites in nanostructured KIT-6 using different methods to obtain efficient acidic catalysts for glycerol acetalization to solketal

Ewa Janiszewska*, Jolanta Kowalska-Kuś, Justyna Wiktorowska, Aldona Jankowska, Agata Tabero, Agnieszka Held, Stanisław Kowalak*

Faculty of Chemistry, Adam Mickiewicz University, Uniwersytetu Poznańskiego 8, 61-614 Poznań, Poland; jolakow@amu.edu.pl (J.K.-K.); juswik1@st.amu.edu.pl (J.W.); aljan@amu.edu.pl (A.J.); agata.tabero@amu.edu.pl (A.T.); awaclaw@amu.edu.pl (A.H.)

* Correspondence: eszym@amu.edu.pl (E.J.), skowalak@amu.edu.pl (S.K.)

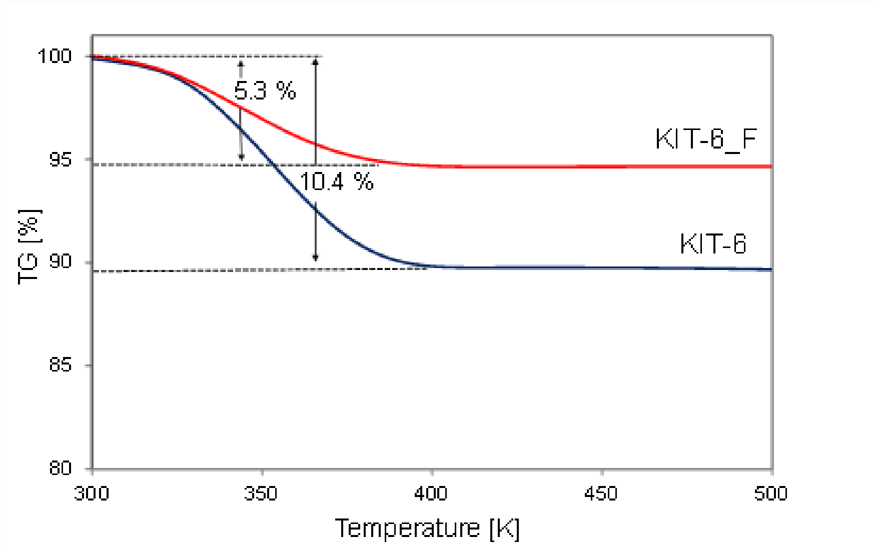

Figure S1. TG analysis of pure silica KIT-6 and NH_4F-modified (KIT-6_F) material.

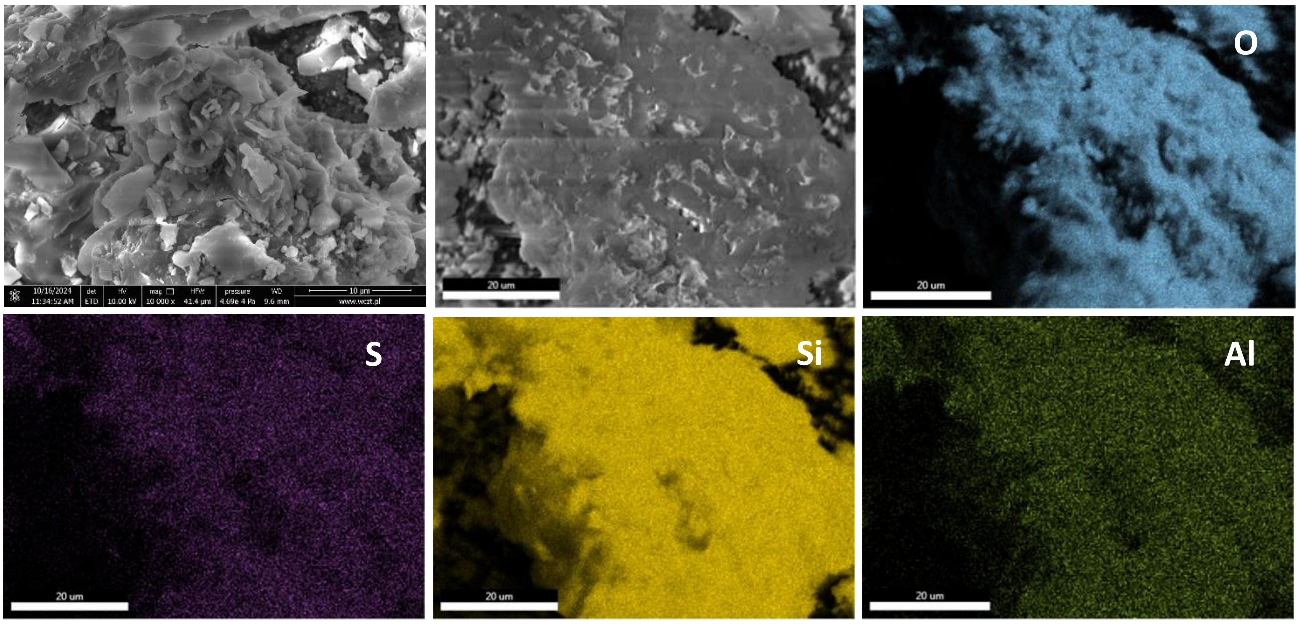

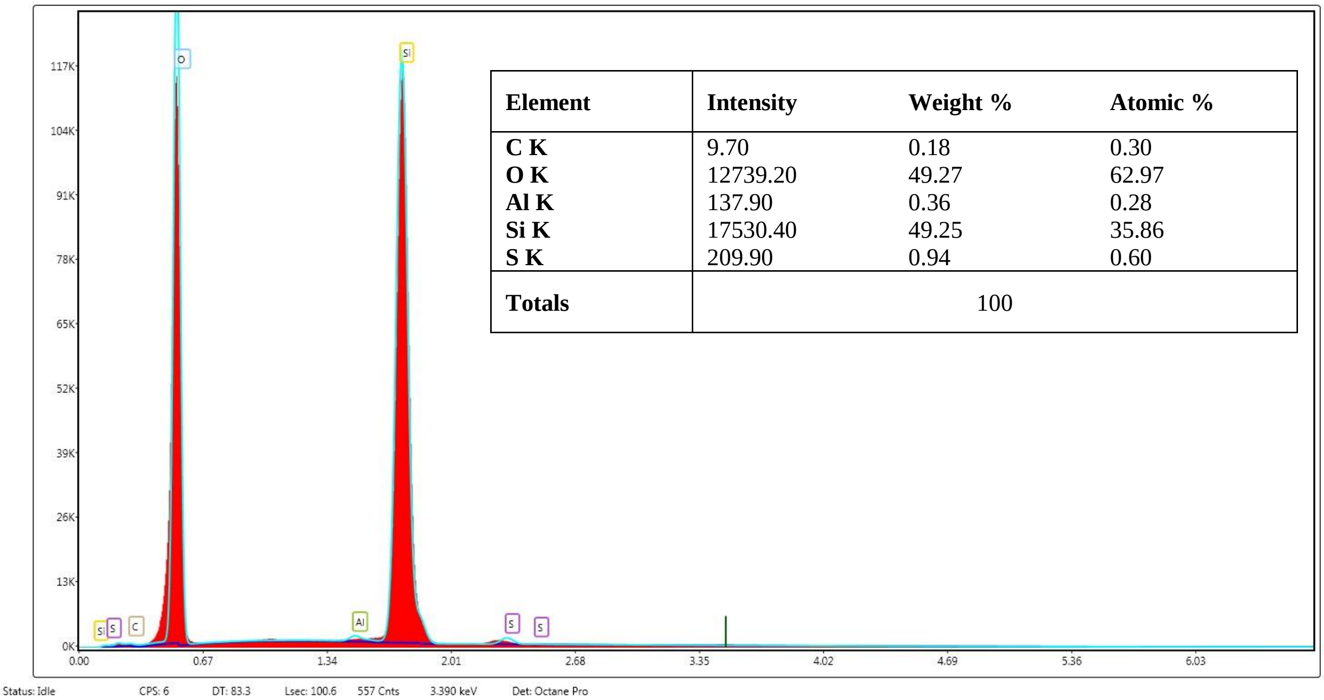

Element	Intensity	Weight %	Atomic %
C K	9.70	0.18	0.30
O K	12739.20	49.27	62.97
Al K	137.90	0.36	0.28
Si K	17530.40	49.25	35.86
S K	209.90	0.94	0.60
Totals		100	

Figure S2. SEM micrographs, elemental mapping, and EDS data of AlKIT-SO_3H material.

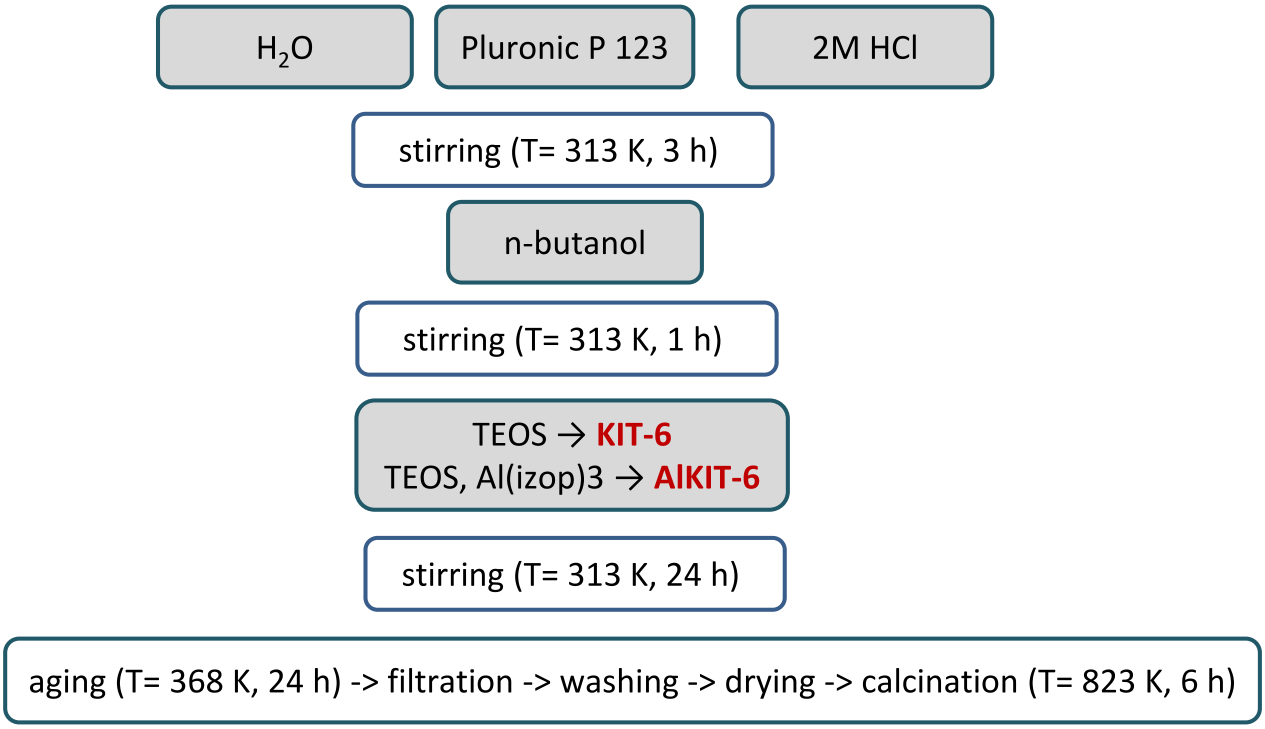

Figure S3. Scheme of KIT-6 and AlKIT-6 synthesis.

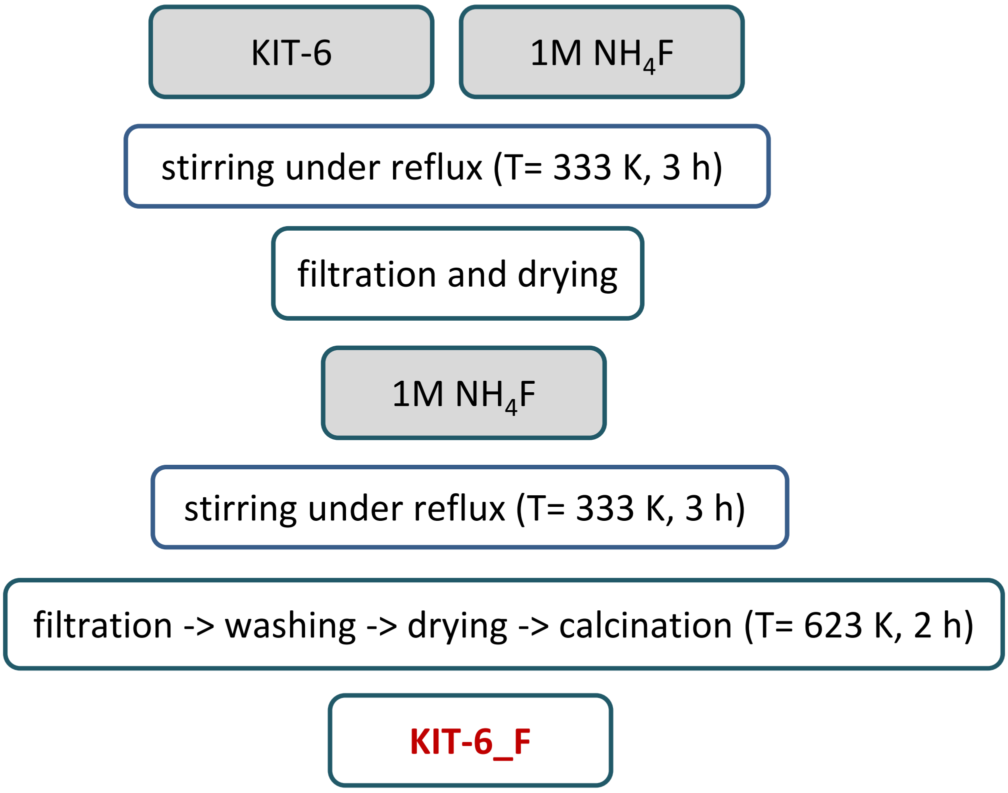

Figure S4. Scheme of KIT-6 modification with NH_4F.

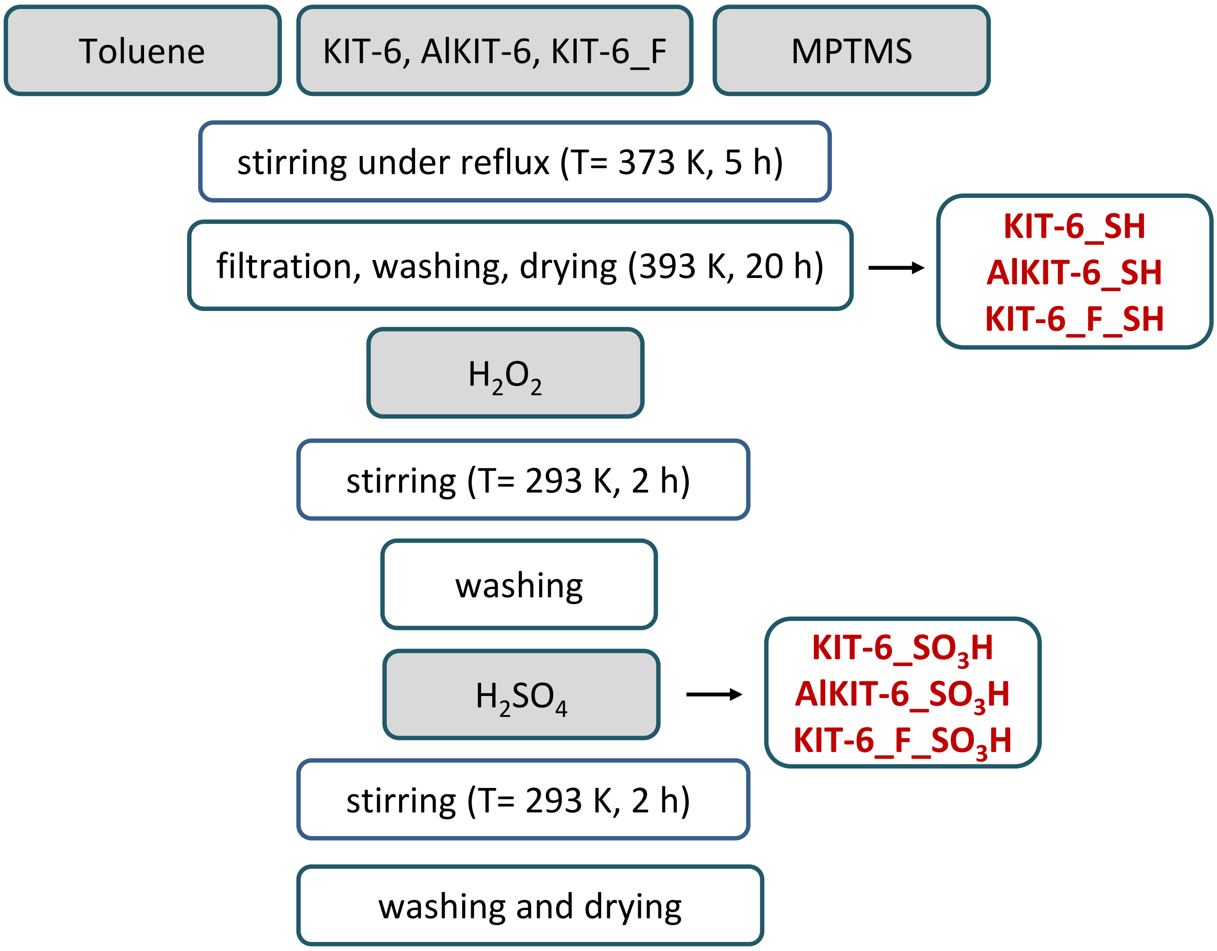

Figure S5. Scheme of grafting of 3-mercaptopropyltrimethoxysilane (MPTMS) and its conversion to sulfonic groups.

Calculation of the catalytic activity results based on the chromatographic data

Conversion of glycerol (%)

$$Glycerol\ conv. = \frac{mol\ of\ glycerol\ converted}{initial\ mol\ of\ glycerol} \times 100 \qquad \text{(Eq. 1)}$$

where mol of glycerol converted = initial mol of glycerol – final mol of glycerol

Selectivity to solketal (%)

$$S\ solketal = \frac{mol\ of\ solketal\ formed}{mol\ of\ glycerol\ converted} \times 100 \qquad \text{(Eq. 2)}$$

Selectivity to isomer (%)

$$S\ isomer = \frac{mol\ of\ isomer\ formed}{mol\ of\ glycerol\ converted} \times 100 \qquad \text{(Eq. 3)}$$

Yield of solketal (%)

$$Ysolketal = \frac{Selecticity\ to\ solketal \times Glycerol\ conv.}{100} \qquad \text{(Eq. 4)}$$

Figure S6. Calculation of the catalytic activity results based on chromatographic data.
